# Shallow shotgun sequencing reduces technical variation in microbiome analysis

**DOI:** 10.1038/s41598-023-33489-1

**Published:** 2023-05-11

**Authors:** Alex J. La Reau, Noah B. Strom, Ellen Filvaroff, Konstantinos Mavrommatis, Tonya L. Ward, Dan Knights

**Affiliations:** 1 Diversigen, Inc., 600 County Road D, West, Suite 8, New Brighton, MN 55112 USA; 2 Bristol Myers Squibb, 1500 Owens St, Suite 600, San Francisco, CA 94158 USA; 3 Department of Computer Science and Engineering, University of Minnesota, Minneapolis, MN 55455 USA; 4 Biotechnology Institute, College of Biological Sciences, University of Minnesota, Minneapolis, MN 55455 USA

**Keywords:** Microbiology, Metagenomics, Microbiome

## Abstract

The microbiome is known to play a role in many human diseases, but identifying key microbes and their functions generally requires large studies due to the vast number of species and genes, and the high levels of intra-individual and inter-individual variation. 16S amplicon sequencing of the rRNA gene is commonly used for large studies due to its comparatively low sequencing cost, but it has poor taxonomic and functional resolution. Deep shotgun sequencing is a more accurate and comprehensive alternative for small studies, but can be cost-prohibitive for biomarker discovery in large populations. Shallow or moderate-depth shotgun metagenomics may serve as a viable alternative to 16S sequencing for large-scale and/or dense longitudinal studies, but only if resolution and reproducibility are comparable. Here we applied both 16S and shallow shotgun stool microbiome sequencing to a cohort of 5 subjects sampled twice daily and weekly, with technical replication at the DNA extraction and the library preparation/sequencing steps, for a total of 80 16S samples and 80 shallow shotgun sequencing samples. We found that shallow shotgun sequencing produced lower technical variation and higher taxonomic resolution than 16S sequencing, at a much lower cost than deep shotgun sequencing. These findings suggest that shallow shotgun sequencing provides a more specific and more reproducible alternative to 16S sequencing for large-scale microbiome studies where costs prohibit deep shotgun sequencing and where bacterial species are expected to have good coverage in whole-genome reference databases.

## Introduction

The human gut microbiome is the community of microbes inhabiting the gastrointestinal tract, and variation in these microbes is known to be associated with numerous human diseases^1,2^. Recent studies have made progress in understanding potential causal roles for microbiome constituents in these disease states^3^. However, these efforts face challenges stemming from the multidimensional and variable nature of the microbiome, with substantial variation occurring between individuals and over time within individuals^4^. In addition to the high amount of biological variation seen in the microbiome, sources of technical variation from sample storage, DNA extraction, library preparation and type of sequencing all affect the microbiome composition determined in any given study^5–8^.

One important consideration in microbiome characterization is whether to perform 16S rRNA gene amplicon or metagenomic sequencing. 16S sequencing has been used widely for over a decade to characterize diverse microbial communities at the taxonomic level and is cost-effective, allowing for very large cohorts with well-powered, complex study designs. However, 16S sequencing lacks the ability to classify microbiomes below the genus level in most cases^9^ and only provides approximate profiles of microbial genes and functions^10–12^. Deep shotgun metagenomic sequencing (DS), defined here by depths greater than 10 million reads per sample, provides detailed characterization of taxonomic composition at the species and strain levels as well as directly observed gene profiles^13,14^. However, it can be cost-prohibitive for large studies and is most often used in biomarker discovery studies with relatively small sample sizes.

An alternative to both 16S amplicon and DS metagenomic sequencing is shallow shotgun metagenomic sequencing (SS)^15^. SS, defined here by depths between 2 and 5 million reads per sample, shows high concordance with DS in both taxonomic and functional gene content at a cost comparable to 16S sequencing^15^. Similar to DS, SS can resolve taxa to the species or even strain levels, can provide directly observed gene profiles, and has been leveraged to perform robust taxonomic and functional characterization of the microbiome in a cohort with dense longitudinal sampling^16^. However, the reproducibility of SS has not been explicitly compared to 16S.

**Figure 1:**
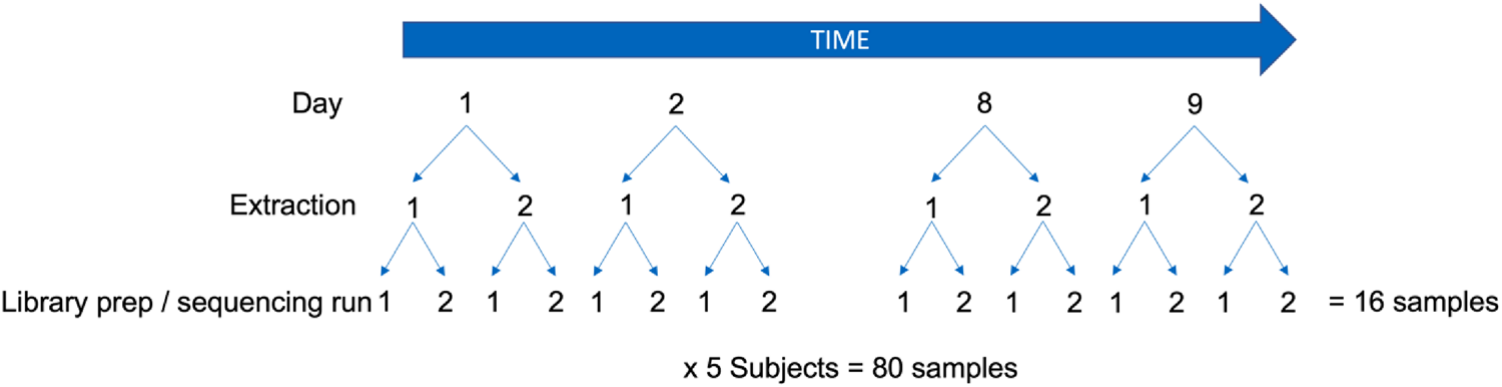
Schematic representation of experimental design. Depicted are the methods used to derive samples from 5 volunteer donors. Biological variation was examined temporally by day/week as well as by subject, while technical variation was examined through replicate extraction (n = 2) and library preparation/sequencing (n = 2).

In order to design and perform robust analyses of microbiome associations in health and disease, it is important to understand the sources of variability in microbiome measurement methods as well as the relative contributions of temporal and interpersonal variability. Here, we sought to compare the efficacy of SS sequencing to 16S amplicon sequencing for recovering species-level taxonomic and functional profiles of the human microbiome, and to assess whether each method can distinguish biological variation from technical variation using an experimental design with nested technical replicates (Fig. 1). Specifically, we aimed to compare interpersonal microbiome variation, daily and weekly intrapersonal microbiome variation, and technical variation resulting from DNA extraction or library preparation and sequencing. Our aim was to explore whether each method could quantify these separate sources of variation, as well as to determine whether 16S or SS had higher technical variation.

## Results

### SS sequencing recovers individualized microbiome profiles with higher precision than 16S sequencing

**Figure 2:**
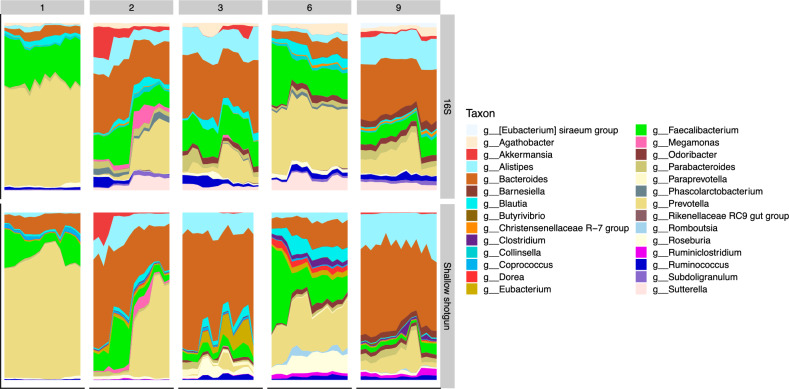
Area plots depicting individualized microbiome profiles at the genus level for the five subjects (denoted by facets on the plot) across their respective sampling periods on the x-axis (days). Plots show relative abundances across sampling for the top 20 most abundant genera detected in 16S amplicon (top) and shallow shotgun metagenomic (bottom) sequencing. Samples were ordered to illustrate changes between consecutive weeks and consecutive days, between DNA extraction replicates within each day, as well as between sequencing run replicates within each extraction. The most resolved taxonomic level for each taxon is plotted. Classifications for 16S were performed using DADA2 against the SILVA database (v132), while classifications for shallow shotgun sequencing were performed using BURST against a custom database of bacterial genomes derived from RefSeq.

To assess changes in the composition of the microbiome within each subject across sampling time points, we first determined the 20 most abundant taxa (regardless of taxonomic level) across subjects for both 16S and SS sequencing separately (Supplementary Table 1). Overall, we found moderate agreement in taxonomic classification for the top 20 taxa between 16S and SS sequencing, with several taxa shared between the two using exact taxonomic string matching, and others matching through inference of shared taxonomy (e.g. species-level assignment falling within a known genus-level assignment) (Supplementary Table 1). To directly compare 16S and SS microbiome profiles, we then plotted the relative abundances of the top 20 most abundant genera across the sampling period within each subject for both 16S and SS. Samples were ordered to illustrate changes between consecutive weeks and consecutive days, between DNA extraction replicates within each day, as well as between sequencing run replicates within each extraction. For both 16S and SS sequencing, we found that microbiome composition within individuals was highly individualized, but that profiles for the same subjects were similar between sequencing types at the genus level (Fig. 2).

When we quantified the degree of concordance between 16S and SS, we found that the relative abundance profiles of shared taxa identified at the genus level were correlated at r = 0.93 and ρ = 0.73 with Pearson and Spearman correlation, respectively, counting absent genera as zeroes (Supplementary Fig. 1).

SS and 16S sequencing varied in the taxonomic resolution of the top 20 most abundant taxa. SS was successful in classifying 14/20 of the top 20 most abundant taxonomic groups to the species level, representing 44.7% mean relative abundance across samples. Conversely, the deepest 16S resolution was at the genus level for the top 20 taxa despite an attempt to assign species-level taxonomy using exact amplicon-sequence-variant (ASV) matching. Similarly, a majority (~ 62.5%) of reads were assigned to the species or strain levels using SS, while only ~ 36% of reads were assigned to the species level using 16S (Supplementary Fig. 2). In both cases, ~ 85% of reads were assigned to at least the genus level.

### SS directly measures functional variation that mirrors taxonomic variation

**Figure 3:**
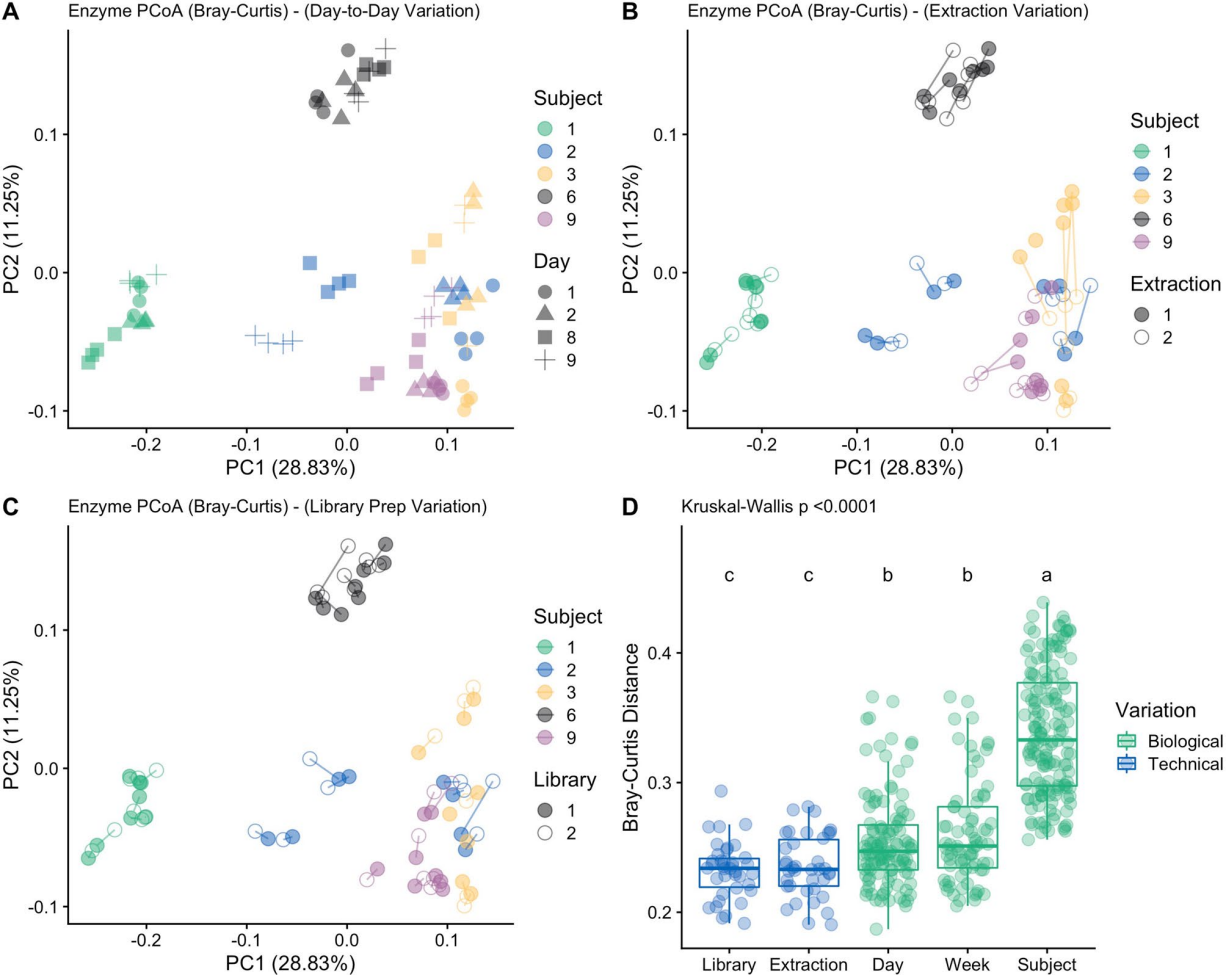
Principal coordinates analysis (PCoA) plot showing the Bray–Curtis dissimilarity of KEGG Enzyme profiles between all samples from SS sequencing. Samples are colored by subject. Percent of variation explained by PC1 and PC2 are shown in parentheses. The same PCoA is plotted showing the clustering of each subject’s samples, depicting (**A**) day-to-day variation, (**B**) extraction replicate variation, and (**C**) library prep replicate variation. Lines connect samples from shared replicates to visualize variation more clearly. (**D**) Comparative sources of microbiome variation depicted using boxplots with all sample data plotted for SS sequencing. Sources of variation are colored by category. Statistical significance letters are annotated above each category from the results of Dunn’s post-hoc test with Benjamini–Hochberg multiple hypothesis correction.

**Figure 4:**
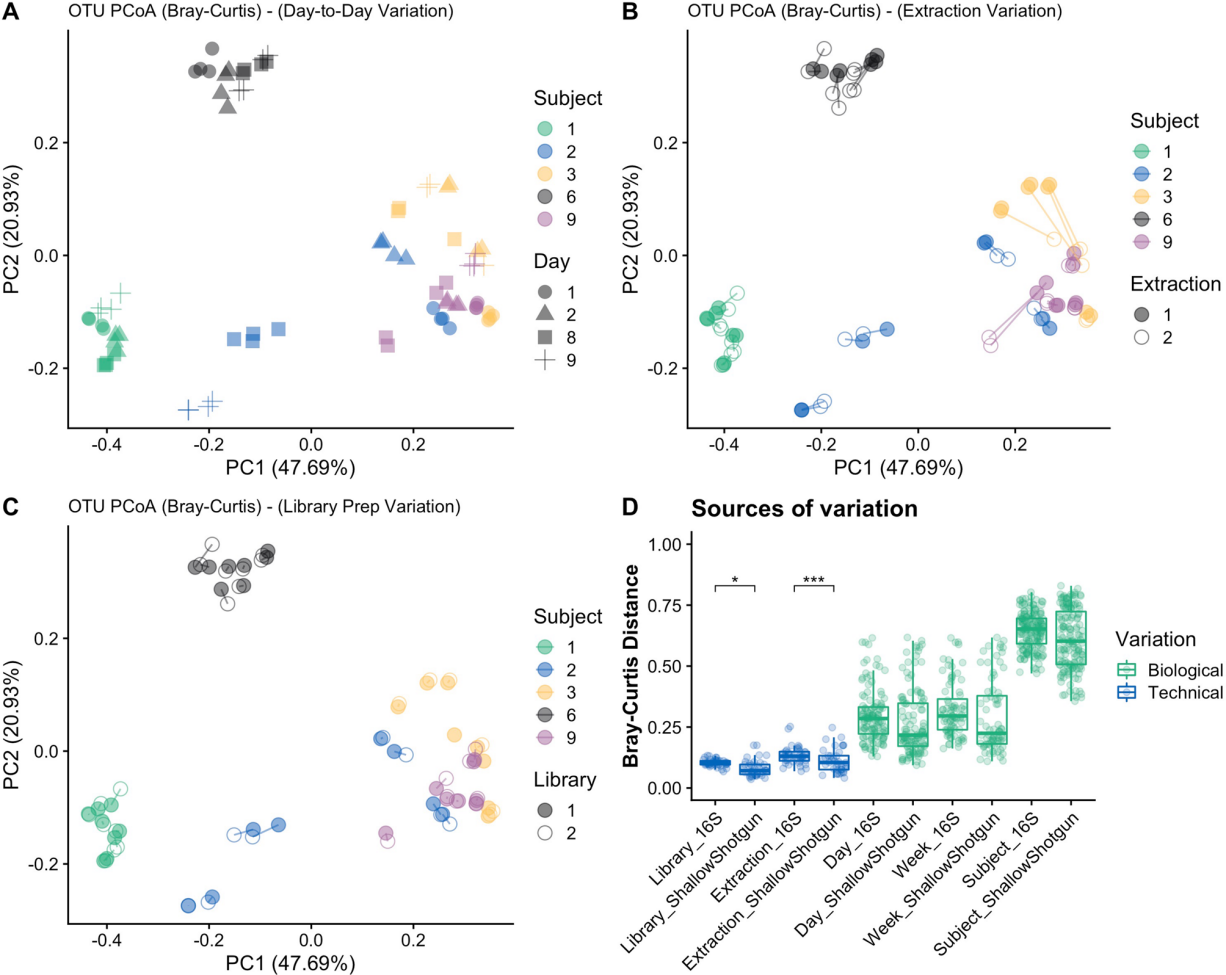
Principal coordinates analysis (PCoA) plot showing the Bray–Curtis dissimilarity of taxa profiles between all samples from SS sequencing. Samples are colored by subject. Percent of variation explained by PC1 and PC2 are shown in parentheses. The same PCoA is plotted showing the clustering of each subject’s samples, depicting (**A**) day-to-day variation, (**B**) extraction replicate variation, and (**C**) library prep replicate variation. Lines connect samples from shared replicates to visualize variation more clearly. (**D**) Comparative sources of microbiome variation depicted using boxplots with all sample data plotted for 16S vs SS sequencing. Sources of variation are colored by category. Statistical significance is annotated above relevant tested groups from the results of t-tests with Benjamini–Hochberg multiple hypothesis correction.

To explore individualized functional composition of the microbiome for each subject, we analyzed inter-subject diversity using Bray–Curtis dissimilarity. Figure 3a–c shows principal coordinate analysis (PCoA) ordinations of KEGG Enzyme Bray–Curtis dissimilarities, highlighting the ability of SS to capture distinct groupings of subjects based on the functional repertoire of their respective microbiomes. To test whether differences between subjects were significant, we first performed a beta dispersion test and found unequal variance in different subjects (“*betadisper*” in the vegan R package; p = 0.001). To accommodate unequal group variances, we performed testing for group differences with PERMANOVA (“*adonis*” in the vegan R package) and found that differences in functional profiles were significant between subjects (PERMANOVA; R = 0.9661, p = 0.001). These results were mirrored at the taxonomic level for SS sequencing (Fig. 4a–c; PERMANOVA: R = 0.9202, p = 0.001) and 16S amplicon sequencing (Supplementary Fig. 3a–c; PERMANOVA: R = 0.8515, p = 0.001). Similarly, we analyzed within-subject functional diversity using three alpha diversity metrics (Shannon index, Chao1 and Observed features). For each metric, group-wise KEGG enzyme alpha diversity differed significantly across subjects (Supplementary Fig. 4a–c; Kruskal–Wallis: p < 0.0001). Results were similar for taxonomic alpha diversity for both 16S (Supplementary Fig. 5a–c) and SS sequencing (Supplementary Fig. 6a–c).

### Technical variation is lower in SS versus 16S sequencing

Our nested sampling design allowed us to compare potential sources of technical variation to each other and to sources of biological variation, using both 16S and SS. We accomplished this comparison by partitioning beta diversity dissimilarities into various categories which isolated one variable at a time: those between DNA extractions on the same sequencing run, subject, and day; those between library preparations of the same DNA extraction, subject, and day; those between consecutive days within the same subject; those between consecutive weeks within the same subject; and those between subjects (Fig. 4a–c).

Overall, we found that sources of technical variation were significantly lower than sources of biological variation at the taxonomic level for both 16S sequencing and SS sequencing (Dunn’s Test, padj < 0.05) (Supplementary Fig. 7). Library prep replicate and DNA extraction replicate variation was lowest, followed by daily and weekly variation within a subject, and finally between-subject variation. In both 16S and SS, all sources of variation were significantly different from one another (Dunn’s Test, padj < 0.05) except for library prep vs extraction variations, as well as daily vs weekly variation. To specifically test our a priori hypothesis that SS had less technical variation than 16S sequencing, we compared these two within both library prep and extraction categories. Consistent with our previous results, we found using two-way ANOVA that sequencing type (i.e. 16S or SS) was significant (p = 0.0116) as well as category (i.e. library prep or extraction; p = 0.005). Furthermore, SS was significantly lower in variation than 16S sequencing for both library prep (Student’s t-test: p = 0.0003) and extraction (Student’s t-test: p = 0.0351) (Fig. 4d). For SS KEGG enzyme profiles, pairwise comparisons of sources of variation were the same as with taxonomic profiles (Fig. 3d).

## Discussion

In this study, we compared the degrees of technical reproducibility and taxonomic precision of 16S amplicon and shallow shotgun metagenomic sequencing for profiling human microbiomes. Overall, we found good general agreement in the most abundant taxa identified through both sequencing methodologies, and the individualized microbiome profiles captured through SS here agree with previous SS work at the taxonomic level for larger sample sizes^16^. As expected, we found that SS recovered species-level classifications to a much greater degree than 16S amplicon sequencing. Interestingly, we found that, of the top 20 most abundant taxa across all subjects, the majority were classified to the species level with SS while none were classified beyond genus level despite an attempt to do so with exact ASV-matching for 16S. While there were ASVs classifiable to the species level, none of them were part of the most abundant taxonomic groups (Supplementary Table 2).

It is known that short regions of the 16S rRNA gene usually are not able to resolve taxa to the species level^9^. An alternative approach would be to use longer portions of the 16 s rRNA gene spanning multiple variable regions. We used the fourth variable region (V4) of the gene, in concordance with the Earth Microbiome Project protocol^17^, which provides good taxonomic coverage but may not provide the highest taxonomic resolution for all taxa. We note that there are also approaches for amplicon sequencing of nearly complete 16S rRNA genes such as through circular-consensus PacBio sequencing^18^ or synthetic long-read sequencing^19^. A direct comparison of these methods to shotgun metagenomics is outside the scope of this study. We expect such long-read methods to provide species-level taxonomic profiling comparable to that of shallow shotgun sequencing, but at the time of writing these approaches are not cost-effective when compared to shallow shotgun sequencing, and they also do not provide direct functional profiling.

We also confirmed the ability of SS metagenomic sequencing to detect unique functional profiles between individuals that mirror those seen at the taxonomic level. We saw similar patterns of beta diversity with directly observed KEGG enzymes and taxonomic profiles. These were both in agreement with the 16S data, suggesting that these subjects harbor both distinct taxonomic and functional profiles, making them distinguishable in beta diversity space with either data type. Taken together with the taxonomic data, these results highlight the utility of SS metagenomics in higher precision taxonomic information than 16S surveys while also offering enhanced resolution of the functional capabilities of the microbiome. Although it is possible to predict functional profiles from 16S sequencing with approximately 80–90% accuracy^11,12,20^, we have previously found that direct observation of functional profiles in shallow shotgun data had higher concordance with functional profiles from deep shotgun metagenomics^15^.

Another major goal was to directly compare the ability of SS and 16S sequencing to discriminate biological variation (day/week of sampling and subject) from technical variation (DNA extraction and sequencing). We have shown previously that amplicon sequencing, including 16S, is subject to certain intractable bias due to the amplification process^21^, yet to our knowledge no one has directly compared the technical reproducibility of 16S sequencing to that of shallow shotgun sequencing. For both types of sequencing, we found that technical variation was significantly lower than biological variation, in agreement with previous work for both 16S amplicon^22^ and DS metagenomic sequencing^8^ separately. However, by directly comparing the same samples sequenced with 16S amplicon and SS sequencing, we found that SS sequencing was subject to significantly lower technical variation than 16S. This was true even though we used methods designed specifically to minimize technical bias in 16S sequencing^21^.

It is important to note that SS sequencing has certain limitations in comparison to 16S. For example, while metagenomics in general is well-suited for use in high-biomass samples (e.g. human feces), it may not be a good substitute for 16S in the characterization of certain low-biomass samples such as blood, urine, or biopsy specimens. Amplicon sequencing also benefits from a better ability to characterize microbial composition in those environments with fewer cultured and sequenced isolate genomes than are available for the human gut, such as soil or water environments^23,24^. There are also cases where DS is preferable to SS: SS can only provide strain-level microbiome profiles for strains with known genomes, cannot be used for de novo metagenome assembly, and relies on species being present in a reference database. As such, while SS is useful in large epidemiology-scale studies for profiling microbiomes with known species and genes, DS will be required for discovering novel strains and genes. For these reasons, there is no question in our minds that deep sequencing is preferred when research funding is not a limiting factor. Finally, we note that our study demonstrated the superiority of shallow shotgun sequencing over 16S sequencing for measuring only a certain kind of biological variation: microbiome variation between individuals.

Overall, we found that shallow shotgun sequencing is a less noisy and more information-rich alternative to 16S sequencing for taxonomic and functional biomarker discovery in large-scale human microbiome studies. In our direct comparison, shallow shotgun sequencing provided more species-level classification of dominant members of the microbiome, and it showed higher reproducibility, with lower technical variation, than 16S sequencing, making it a reasonable choice for large-scale studies and meta-analyses.

## Methods

### Participant selection and sample collection

Informed consent was obtained for five adult volunteers. The study protocols were reviewed and approved by the Advarra Institutional Review Board (Advarra, Inc., Columbia, MD). All analyses were performed according to the relevant guidelines and regulations. Fecal collection was completed by self-sampling, which has proven to be highly successful in previous work^25^. Briefly, participants each collected a small quantity of fecal material into 90% ethanol using a sterile swab once per day during each day of the planned collection period. Subjects collected one sample to begin their sampling cycle (day 1), with another sample taken on day 2. Subsequent samples were taken exactly one week after the first samples (days 8 and 9). Participants were given postage-paid pre-addressed boxes and asked to drop samples in the US postal service mail to return samples to Diversigen for processing after all four samples were collected. After shipping, all samples were stored at − 80 °C prior to processing.

### DNA extraction, library preparation and sequencing (16S amplicon)

Samples were extracted using a QIAamp PowerFecal DNA Kit (QIAGEN, Germantown, MD, USA) automated for high throughput on a QiaCube, with bead beating in 0.1 mm glass bead plates. Samples were then quantified via qPCR using primers for hypervariable region 4 (V4) (515f/806r) of the 16S rRNA gene. Libraries were prepared using a protocol derived from previous methods^21^ using KAPA HiFi Polymerase to amplify the V4 region (515f/806r). Samples were indexed using Illumina Unique Dual Indexes (UDI), followed by pooling of libraries. The resulting pooled libraries were denatured with NaOH, diluted to a loading concentration of 8 pM in HT1 buffer (Illumina Inc., San Diego, CA, USA), and spiked with 15% PhiX. The final libraries were sequenced on an Illumina MiSeq instrument using paired-end 2 × 250 reads and the MiSeq Reagent Kit v3 (Illumina). Sequences were demultiplexed on the sequencer and then converted to FASTQ files using *bcl2fastq* (Illumina). DNA sequences were filtered for low quality (Q-Score < 20) and length (< 50 bp), and adapter sequences were trimmed using *cutadapt*^26^.

### DNA extraction, library preparation and sequencing (shallow shotgun metagenomics)

Samples were extracted in the same manner as above for amplicon sequencing, followed by quantification using a Quant-iT PicoGreen dsDNA assay kit (Thermo Fisher Scientific, Waltham, MA, USA), with fluorescence measured on a TECAN plate reader (Tecan Group Ltd., Männedorf, Switzerland). Samples were indexed using Illumina Unique Dual Indexes (UDI), followed by pooling of libraries. Libraries were pooled, followed by SPRI bead purification and concentration using SpeedBead Magnetic Carboxylate Modified Particles (GE Healthcare Life Sciences). The resulting pooled libraries were denatured with NaOH, diluted to a loading concentration of 1.8 pM in Illumina’s HT1 buffer, and spiked with 2% PhiX. Shotgun metagenomic sequencing was performed on an Illumina NextSeq 500 instrument using a NextSeq 500/550 High Output 150 cycle kit (1 × 145 bp reads). Sequence reads were demultiplexed and quality filtered in the same manner as above for amplicon sequencing. Finally, FASTQ files were merged and converted into a single FASTA using *SHI7*^27^. All sequences were trimmed to a maximum length of 100 bp prior to alignment.

### Sequence alignment and annotation (16S amplicon)

Amplicon Sequence Variants (ASVs) were determined using DADA2 (v1.12)^28^ with default parameters except where specified. Briefly, *filterAndTrim(…truncLen* = *c(240,150))* was run to trim low-quality tails from sequence reads, followed by *learnErrors* to learn error rates of the full dataset. ASVs were inferred using the *dada* algorithm, and reads were then merged using *mergePairs*. Chimeras were removed using *removeBimeraDenovo*. Finally, ASVs were assigned taxonomies via classification to the Silva reference database (v132)^29,30^ using *assignTaxonomy,* and, where possible, species level assignments using exact matching were made using *addSpecies*. ASVs with identical taxonomic assignments in the ASV table were collapsed to create a taxa table. This collapsed taxa table was used in all downstream analyses.

### Sequence alignment and annotation (shallow shotgun metagenomics)

DNA sequences were aligned to a curated database containing all representative genomes in RefSeq (release 81) for bacteria^31^, with additional manually curated strains (i.e. Diversigen’s Venti database). Alignments were made at 97% identity against all reference genomes. Every input sequence was compared to every reference sequence in Venti using fully gapped alignment with BURST^32^. Ties were broken by minimizing the overall number of unique Operational Taxonomic Units (OTUs) based on taxonomic assignments (i.e. “CAPITALIST” method in BURST). Specifically, each input sequence was assigned the lowest common ancestor that was consistent across at least 80% of all reference sequences tied for best hit. The number of counts for each OTU was normalized to the average genome length. OTUs accounting for less than one millionth of all species-level markers and those with less than 0.01% of their unique genome regions covered (and < 1% of the whole genome) were discarded. Samples with fewer than 10,000 sequences mapping to the database were also discarded. Count data was then converted to relative abundance for each sample. The normalized and filtered tables were used for all downstream analyses. For functional annotation, Kyoto Encyclopedia of Genes and Genomes Orthology groups (KEGG KOs)^33–35^ were observed directly using alignment at 97% identity against a gene database derived from the strain database used above. The KO table was used to derive the downstream KEGG Enzyme table by collapsing KOs to the enzyme level, followed by conversion to relative abundances. This KEGG enzyme table was used for all downstream analyses.

### Diversity metrics calculations, statistical analyses and visualization

Alpha diversity (Chao1, Shannon index and Observed features) and beta diversity (Bray–Curtis dissimilarity) of taxa and functions were calculated using the R^36^ package vegan (v2.6-4)^37^, using the ASV/OTU/KEGG Enzyme tables rarified to the lowest sample depth for their respective sequencing types (amplicon vs. shallow shotgun). To assess differences in alpha diversity between subjects, the nonparametric Kruskal–Wallis one-way analysis of variance followed by Dunn’s post-hoc test were used. False discovery rate (FDR) correction was used to account for multiple hypothesis testing. Differences in beta diversity between groups of interest was assessed via multivariate homogeneity of groups dispersions, followed by permutational multivariate analysis of variance (PERMANOVA) as implemented in vegan’s *betadisper* and *adonis* functions, respectively. To quantify the magnitude of variation from different sources, the Bray–Curtis dissimilarity matrix was subset to include only samples for each given source of interest as follows: (1) technical variation caused by DNA extraction of the same sample, (2) technical variation caused by the same extraction replicate prepped into separate libraries and run on separate sequencing runs, (3) biological variation caused by day of sampling, (4) biological variation caused by week of sampling, and (5) biological variation caused by subject-to-subject variation. Differences in variation by source were assessed using Kruskal–Wallis followed by Dunn’s post-hoc test. Specific differences in technical variation between 16S and shallow shotgun sequencing were assessed using two-way ANOVA followed by student’s t-tests. All visualizations were generated using the R package ggplot2 (v3.3.6)^38^.

### Supplementary Information

Supplementary Legends.

Supplementary Figures.

Supplementary Table 1.

Supplementary Table 2.

## Data Availability

The datasets generated and analyzed during the current study are available in the NCBI SRA repository for both shotgun metagenomic and 16S rRNA gene amplicon data under BioProject accession number PRJNA917645.
