# Author Correction: Shallow shotgun sequencing reduces technical variation in microbiome analysis

**DOI:** 10.1038/s41598-024-56475-7

**Published:** 2024-03-13

**Authors:** Alex J. La Reau, Noah B. Strom, Ellen Filvaroff, Konstantinos Mavrommatis, Tonya L. Ward, Dan Knights

**Affiliations:** 1 Diversigen, Inc., 600 County Road D, West, Suite 8, New Brighton, MN 55112 USA; 2 Bristol Myers Squibb, 1500 Owens St, Suite 600, San Francisco, CA 94158 USA; 3 Department of Computer Science and Engineering, University of Minnesota, Minneapolis, MN 55455 USA; 4 Biotechnology Institute, College of Biological Sciences, University of Minnesota, Minneapolis, MN 55455 USA

Correction to: *Scientific Reports* 10.1038/s41598-023-33489-1, published online 11 May 2023

The original version of this Article contained an error in Figure S7 of the ‘Supplementary Figures’ file, where the labels ‘16S’ and ‘Shallow shotgun’ were swapped.

The incorrect Supplementary file can be found below.

The original Article has been corrected.

### Supplementary Information

Supplementary Figures.

