# Supplementary material for: Shallow shotgun sequencing reduces technical variation in microbiome analysis
Source: Sci Rep. 2023 May 11;13:7668. doi: 10.1038/s41598-023-33489-1 (PMC10175443; doi:10.1038/s41598-023-33489-1)
Supplement: Supplementary file 1 — Supplementary Legends. [file 41598_2023_33489_MOESM1_ESM.docx]

**Supplementary Figure 1.** Correlation scatterplot at the genus level for relative abundances of shared genera between paired 16S and SS sequencing. Results of statistical correlation testing using Pearson and Spearman tests are annotated as plot subtitles.

**Supplementary Figure 2.** Stacked barplots showing the proportion of sequencing reads assigned to each bacterial taxonomic level using both 16S (left) and SS (right) sequencing. Proportions represent the fraction of total reads successfully mapped to respective reference databases.

**Supplementary Figure 3.** PCoA plots showing the Bray-Curtis dissimilarity between all samples using the ASV relative abundance table as input, depicting (A) day-to-day variation, (B) extraction replicate variation, and (C) library prep replicate variation. Lines connect samples from shared replicates to visualize variation more clearly. Samples are colored by subject, and respective technical replicates for each plot are depicted using filled circles for replicate 1 and unfilled circles for replicate 2. Percent of variation explained by PC1 and PC2 are shown in parentheses.

**Supplementary Figure 4.** Boxplots showing differences in alpha diversity metrics (Shannon, Chao1 and Observed features) at the KEGG Enzyme level from SS sequencing data. Each plot is colored by subject, with the result of statistical testing annotated on each plot. Different lowercase letters indicate significant group differences based on Dunn's test.

**Supplementary Figure 5.** Boxplots showing differences in alpha diversity metrics (Shannon, Chao1 and Observed features) at the ASV level from 16S sequencing data. Each plot is colored by subject, with the result of statistical testing annotated on each plot. Different lowercase letters indicate significant group differences based on Dunn's test.

**Supplementary Figure 6.** Boxplots showing differences in alpha diversity metrics (Shannon, Chao1 and Observed features) at the OTU level from SS sequencing data. Each plot is colored by subject, with the result of statistical testing annotated on each plot. Different lowercase letters indicate significant group differences based on Dunn's test.

**Supplementary Figure 7.** Comparative sources of microbiome variation depicted using boxplots with all sample data plotted for 16S (top) and SS (bottom) sequencing. Sources of variation are colored by category. Statistical significance letters are annotated above each category from the results of Dunn’s post-hoc test with Benjamini-Hochberg multiple hypothesis correction.
