# Supplementary material for: Shallow shotgun sequencing reduces technical variation in microbiome analysis
Source: Sci Rep. 2023 May 11;13:7668. doi: 10.1038/s41598-023-33489-1 (PMC10175443; doi:10.1038/s41598-023-33489-1)
Supplement: Supplementary file 2 — Supplementary Figures. [file 41598_2023_33489_MOESM2_ESM.docx]

**Supplementary Figure 1**

**Supplementary Figure 2**

**Supplementary Figure 3**

**Supplementary Figure 4**

**Supplementary Figure 5**

**Supplementary Figure 6**

**Supplementary Figure 7**
